# Estimating the Effective Population Size Across Space and Time in the Critically Endangered Western Chimpanzee in Guinea‐Bissau: Challenges and Implications for Conservation Management

**DOI:** 10.1111/eva.70162

**Published:** 2025-10-10

**Authors:** Maria Joana Ferreira da Silva, Filipa Borges, Federica Gerini, Rui M. Sá, Francisco Silva, Tiago Maié, Germán Hernández‐Alonso, Jazmín Ramos‐Madrigal, Shyam Gopalakrishnan, Isa Aleixo‐Pais, Saidil Lamine Djaló, Nelson Fernandes, Idrissa Camará, Aissa Regalla, Catarina Casanova, Mafalda Costa, Ivo Colmonero‐Costeira, Carlos Rodríguez Fernandes, Lounès Chikhi, Tânia Minhós, Michael W. Bruford

**Affiliations:** ^1^ CIBIO – Centro de Investigação em Biodiversidade e Recursos Genéticos Universidade do Porto Vairão Portugal; ^2^ BIOPOLIS Program in Genomics, Biodiversity and Land Planning CIBIO Vairão Portugal; ^3^ School of Biosciences Cardiff University Cardiff Wales, UK; ^4^ Centre for Research in Anthropology (CRIA‐NOVA FCSH/IN2PAST) Edifício 4 – Iscte Conhecimento e Inovação Lisboa Portugal; ^5^ Anthropology Department, School of Social Sciences and Humanities Universidade Nova de Lisboa (NOVA FCSH) Lisboa Portugal; ^6^ Centre for Ecology and Conservation University of Exeter Cornwall UK; ^7^ Instituto Gulbenkian de Ciência Oeiras Portugal; ^8^ Dipartimento di Biologia dell'Università di Pisa Pisa Italy; ^9^ Centre for Public Administration and Public Policies Institute of Social and Political Sciences, Universidade de Lisboa Lisbon Portugal; ^10^ CE3C – Centre for Ecology, Evolution and Environmental Changes & CHANGE – Global Change and Sustainability Institute, Departamento de Biologia, Faculdade de Ciências Universidade de Lisboa Lisboa Portugal; ^11^ Section for Hologenomics The Globe Institute, University of Copenhagen Copenhagen Denmark; ^12^ Abu Village Ganogo Island Guinea‐Bissau; ^13^ Anghôr Village Orango Island Guinea‐Bissau; ^14^ Instituto para a Biodiversidade e Áreas Protegidas (IBAP) Bissau Guiné‐Bissau; ^15^ CIAS, Research Centre for Anthropology and Health, Department of Life Sciences Universidade de Coimbra Coimbra Portugal; ^16^ Instituto Superior de Ciências Sociais e Políticas Universidade de Lisboa Lisboa Portugal; ^17^ Faculdade de Psicologia Universidade de Lisboa, Alameda da Universidade Lisboa Portugal; ^18^ Centre de Recherche sur la Biodiversité et l'Environnement (CRBE UMR5300) Université de Toulouse, Toulouse INP, CNRS, IRD, CRBE Toulouse France

**Keywords:** Anthropogenic landscapes, Demographic history, Genetic diversity, Great ape, microsatellite loci, *Pan troglodytes verus*, Whole‐genome sequences

## Abstract

The western chimpanzee ( *Pan troglodytes verus* ) is a Critically Endangered taxon. In Guinea‐Bissau (GB), the subspecies is increasingly threatened, but there is a lack of understanding regarding the degree of genetic threat faced by populations. This hinders the development of targeted conservation strategies and the prioritization of efforts by national agencies. In this study, we use microsatellite data from four parks located in southern GB and five whole‐genome sequences to estimate the effective population size (*N*_*e*_) and infer the recent and ancient demographic history of populations using different methods. We also aim to integrate the different *N*_*e*_ estimates to improve our understanding of the evolutionary history and current demography of this great ape and to discuss the strengths and limitations of each estimator and their complementarity in informing conservation decisions. Results from the PSMC method suggest a large ancestral *N*_e_, likely due to ancient structure over the whole subspecies distribution until approximately 10,000–15,000 years ago. After that, a change in connectivity, a real decrease in size, or a combination of both occurred, which reduced the then still large ancestral population to a smaller size (MSVAR: ~10,000 decreasing to 1,000–6,000 breeding individuals), possibly indicating a fragmentation into coastal and inland subpopulations. In the most recent past, contemporary *N*_e_ is close to 500 (GONE: 395–583, NeEstimator: 107–549), suggesting a high risk of extinction. The populations located at the coastal parks may have been small or isolated for several generations and are at higher risk, whereas the ones located inland exhibit higher long‐term *N*_e_ and can be considered a stronghold for chimpanzee conservation. Through combining different types of molecular markers and analytical methodologies, we tried to overcome the limitations of obtaining high‐quality DNA samples from wild threatened populations and estimated *N*_e_ at different temporal and spatial scales, which is crucial information to make informed conservation decisions at local and regional scales.

## 1 Introduction

The concept of effective population size (*N*_*e*_) is central in evolutionary and conservation biology and has important practical applications in conservation management (Frankham et al. 2010; Hoban et al. 2022; Waples 2022). *N*_*e*_ is considered as probably the most important metric to understand and predict both the populations' short‐term risk of extinction by inbreeding depression and their long‐term potential to adapt to environmental changes (Hoban et al. 2020, 2022). *N*_*e*_ is also one of the best‐studied metrics for applying minimal viable population thresholds and identifying populations of conservation concern (Frankham 2005; Jamieson and Allendorf 2012; Frankham et al. 2014) and has many practical applications in wildlife management and conservation planning (Luikart et al. 2010; O'Brien et al. 2022; Waples 2022, 2024). For instance, *N*_*e*_ has been considered one of the four genetic *Essential Biodiversity Variables* (summary measures of biodiversity), which are designed to monitor changes in biodiversity over time and space (Hoban et al. 2022).

Nevertheless, the estimation and practical integration of the *N*_*e*_ parameter into conservation management and policies is advancing slowly, even in regions with high biodiversity and for iconic and endangered species (Bertola et al. 2024). This is related to several factors concerning feasibility and low financial resources to estimate *N*_*e*_ (e.g., Bertola et al. 2024; Waples 2022). Moreover, in the context of conserving threatened species, while the census size (*N*_*c*_) may have direct relevance, the importance of *N*_*e*_ is less immediately apparent. Decades of research have shown repeatedly that *N*_*e*_ and *N*_*c*_ are not only distinct but may have nearly opposite trends under some models (Wakeley 1999; Mazet et al. 2016, Appendix S1), and thus, it may be relevant to estimate both parameters. Furthermore, although *N*_*e*_ seems easy to understand and to compute using genetic data (Allendorf et al. 2010), it is perhaps one of the most difficult and error‐inducing concepts to grasp in population genetics. One reason for this is that *N*_*e*_ is a single number that aims at summarizing a usually highly complex situation, whether we are interested in the demographic history or recent dynamics of a species (Chikhi et al. 2010; Chikhi et al. 2018; Wakeley 1999; Waples 2022).

*N*_*e*_ quantifies the rate of genetic change (e.g., drift of allele frequencies) of real populations in reference to the Wright–Fisher (WF) idealized population (Wang et al. 2016). *N*_*e*_ is thus the size of an idealized WF population with the same properties of genetic drift as the real (more complex) population under consideration (Wang et al. 2016). Different types of *N*_*e*_ can be identified depending on the property of interest that *N*_*e*_ is supposed to summarize (e.g., inbreeding effective size, *N*_*eI*_, or the variance effective size, *N*_*eV*_, Ryman et al. 2019). The concept of coalescent *N*_*e*_ was also defined to identify the *N*_*e*_ that can explain the patterns of diversity observed in present‐day populations under simple demographic models, typically assuming panmixia over long periods of time (i.e., reflecting the population's evolutionary history). This concept has itself been extended by allowing *N*_*e*_ to change through time. Note that under the latter case, there is not one *N*_*e*_ but rather a succession of *N*_*e*_ values, which may thus lead to apparent contradictions between methods estimating one *N*_*e*_ and those estimating a succession of *N*_*e*_ values.

Under a standard constant‐size WF model, all the different *N*_*e*_ concepts are expected to be the same. However, this is not necessarily the case in real‐world situations, where populations are rarely panmictic or at mutation‐drift equilibrium. Real populations have likely gone through complex demographic histories involving expansions and contractions related to environmental and/or habitat connectivity changes (Wang et al. 2016; Ryman et al. 2019). In addition, theoretical work suggests that there may be demographic models for which some *N*_*e*_ cannot be defined (Sjödin et al. 2005). The point we wish to make here is that depending on the research questions asked, one may obtain very different answers. The fact that we obtain different values should be seen as an indication that the species of interest may not be easily summarized by a single *N*_*e*_ value and that the different estimates obtained might all be useful for devising conservation strategies that account for both the ongoing dynamics of the species and its demographic history.

**FIGURE 1:**
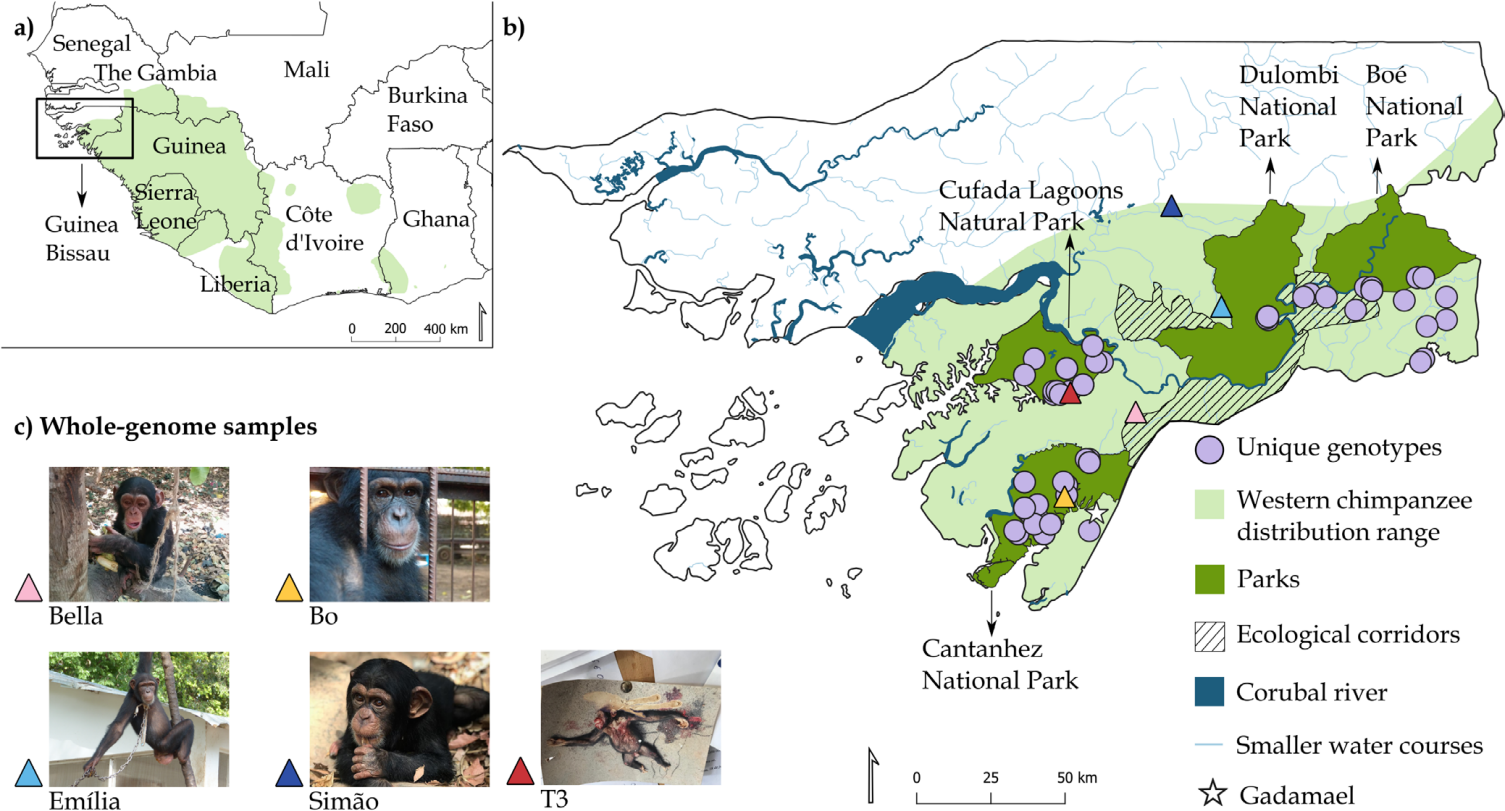
(a) Distribution of the western chimpanzee ( *Pan troglodytes verus* ) in West Africa and (b) the location of the study area in Guinea‐Bissau (represented in light green). Unique genotypes for 10 microsatellite loci (represented by purple circles, *N* = 143) were obtained from fecal samples collected non‐invasively in four parks located in southern Guinea‐Bissau (represented in dark green) and encompassing the national range of chimpanzees—Cufada Lagoons Natural Park (CLNP), Cantanhez National Park (CNP), Dulombi National Park (DNP) and Boé National Park (BNP). The location of ecological corridors is also indicated (in stripped lines). Gadamael area (referred to in Table 1) is also mapped and represented as a white star. (c) Pictures show the four confiscated chimpanzees and one road‐killed individual that were sampled to generate whole‐genome sequencing data. Blood samples were drawn from the confiscated individuals during placement in a sanctuary abroad. One muscle sample was obtained from one roadkill, minutes after fatality. The location of confiscated individuals represented by triangles in the map in (b) is estimated and reflects information on the individual's origin obtained from national authorities (e.g., Bo from CNP) or where individuals were found in private premises. Photo credits by authors and H. Foito (European‐Union Embassy, Bissau), P. Medro (Vetnatura) and L. Espirito Santo.

The western chimpanzee ( *Pan troglodytes verus* , Schwarz 1934) is one of the four currently recognized subspecies of chimpanzees *P. troglodytes* . Its range extends from Senegal in the west to Ghana in the east (Figure 1a) (Campbell and Houngbedji 2015; Ginn et al. 2013; IUCN SSC Primate Specialist Group 2020). Western chimpanzees live in communities of between 12 and 84 individuals (Boesch and Boesch‐Achermann 2000; Matsuzawa et al. 2011) and have a relatively late onset of reproduction (i.e., > 10 years for females), only one offspring per pregnancy, and an interbirth interval of around 5–6 years (reviewed in Thompson 2013). Early life mortality rates vary across studied populations, but there is a tendency for fewer than half of the individuals to survive to the age of the first reproduction (Hill et al. 2001).

The subspecies has been classified as Critically Endangered by the International Union for Conservation of Nature (IUCN) (Humle et al. 2016). The global population of the western chimpanzee is estimated to have decreased by 80% between 1990 and 2014 (Kühl et al. 2017) and to have reached a size between 17,577 and 96,564 individuals (95% Confidence Interval (CI), Heinicke, Boesch, et al. 2019). The number of individuals is expected to decrease in the next decades given the general lack of formal protection of its populations and high rates of habitat loss predicted for West Africa (Heinicke, Boesch, et al. 2019; Palminteri et al. 2018). The western chimpanzee is also threatened by hunting to supply the trade of wild meat and body parts, live animals, and by diseases (Humle et al. 2016; IUCN SSC Primate Specialist Group 2020; The Arcus Foundation 2020; Sá et al. 2012). When compared to the other *P. troglodytes* subspecies, the western chimpanzee has low genetic diversity and two recent studies have suggested that *N*_*e*_ could be in the order of 17,378 effective number of breeders (de Manuel et al. 2016; Fontsere et al. 2022), even if this number should be interpreted with caution (Steux et al. 2024).

In Guinea‐Bissau (GB, area: 36,125 km^2^, human population: 2.08 million), the western chimpanzee (*Dari*, in GB Creole) occurs mainly south of the Corubal River (Bersacola et al. 2018; Carvalho et al. 2013) (Figure 1b). Chimpanzees were erroneously declared extinct in the country and were rediscovered in the 1990s (Gippoliti and Dell'Omo 1996; Gippoliti and Dell'Omo 2003). Identified conservation threats include habitat loss and fragmentation, retaliatory killing during crop‐foraging, and harvest of young live individuals for illegal trade that may imply killing adult individuals (Hockings and Sousa 2013; Ferreira da Silva and Regalla 2025; Ferreira da Silva, Minhós, et al. 2021). Commerce of skins for traditional medicine practices was observed, although the national origin of these specimens has not been confirmed (Sá et al. 2012). Contrary to what happens in other countries (e.g., Côte d'Ivoire; Caspary et al. 2001), the trade of chimpanzee meat does not seem to occur in GB (Ferreira da Silva, Camará, et al. 2021; Minhós et al. 2013; van Laar 2010), probably because it is considered non‐edible by locals due to chimpanzees' high resemblance to humans (Amador et al. 2014; Gippoliti and Dell'Omo 2003; Karibuhoye 2004; Sousa et al. 2017). Chimpanzees in Cantanhez National Park (CNP, Figure 1) may be affected by the propagation of diseases such as leprosy ( *Mycobacterium leprae* ) (Hockings et al. 2021). Past studies reporting a high prevalence of parasites shared with humans suggest that habitat disturbance plays a role in the transmission and persistence of pathogens (Sá et al. 2013).

GB is an important area for the conservation of the western chimpanzee. Specifically, (i) the coastal areas of GB together with the ones in the Republic of Guinea are considered a priority region (Kormos and Boesch 2003; IUCN SSC Primate Specialist Group 2020), (ii) the protected areas of CNP, Dulombi National Park (DNP), and Boé National Park (BNP) (Figure 1) are considered areas of high conservation value (Heinicke, Boesch, et al. 2019), and (iii) the Boé region, where BNP is located, is one of eight sites across the subspecies distribution that is classified as exceptionally stable or of high density (Heinicke, Mundry, et al. 2019). However, national conservation management needs some important improvements, namely baseline estimates of demographic parameters (e.g., population size) and of genetic diversity; such information could be used to inform the prioritization of areas to conserve (Ferreira da Silva and Bruford 2017) and the long‐term viability of local populations (IUCN SSC Primate Specialist Group 2020).

The overall western chimpanzee population in GB has been suggested to be between 600 and 1000 (Gippoliti and Dell'Omo 2003) or 1908 individuals (Heinicke, Boesch, et al. 2019) but improved representative surveys have been recommended given the large confidence intervals of estimates (e.g., 95% CI: 923–6,121 in Heinicke, Boesch, et al. 2019). The size of local populations has been evaluated for most of the parks where chimpanzees occur using various indirect methods (Table 1). Although the estimates from the different studies cannot be compared directly, Cufada Lagoons Natural Park (CLNP) is highlighted as the population with the lowest density, whereas the BNP is the one displaying the topmost density (reaching > 6 individuals per km^2^, Table 1). Currently, there is no size or density assessment for DNP or for other populations outside areas with formal protection, such as ecological corridors (but see the exception of Gadamael, Table 1, Figure 1b), although the proportion of chimpanzees living outside a park in GB may reach 35% (Heinicke, Boesch, et al. 2019). Genetic tools have not yet been applied to inform the conservation of the western chimpanzees in GB (but see Borges 2017; Gerini 2018). Nevertheless, unravelling the evolutionary and demographic history, genetic diversity and the historical and current connectivity between populations is of paramount importance to understand how chimpanzees in GB have been responding to environmental changes over time, which in turn will inform conservation strategies in the face of ongoing and future changes.

**TABLE 1:** Compilation of results from previous studies estimating the density and number of individuals of local populations of western chimpanzees ( *Pan troglodytes verus* ) in Guinea‐Bissau.

Site	Study reference	Method	Estimated density (ind./km^2^)	Estimated number of individuals
Cantanhez National Park	Sousa (2009)	Nest count method for density estimation	1,937 and 2,340	*2,070 and 2,454
	Torres et al. (2010)	Modeling	MI	376 and 2,632
	Hockings and Sousa (2013)	MI	3.00	MI
Cufada Lagoons Natural Park	Carvalho et al. (2013)	Nest count method for density estimation	0.22	137 (95% C.I.: 51–390)
	Sousa et al. (2014)	Marked nest count method	0.79 (95% CI: 0.61–1.04)	300 (95% C.I.: 230–390)
Gadamael	Sousa (2009)	Nest count method for density estimation	0.897	33
Boé National Park	Schwarz et al. (2007)	Interviews	MI	710
	Dias et al. (2019)++	Nest count method for density estimation	0.38	18
	Wenceslau (2014)	Nest count method for density estimation	1.8	MI
	Binczik et al. (2017)	Standing crop nest count method	0.77 (95% CI: 0.45–1.34)	1,465 to 4,415
	Heinicke, Boesch, et al. (2019)	Modeling	6.76	MI
Overall population	Heinicke, Boesch, et al. (2019)	Modeling	MI	1,908 (95% CI: 923–6,121)

*Note:* The geographic location of sites within the country is indicated in Figure 1. The study areas of most studies are National or Natural Parks, except for Gadamael, which is an area not formally protected. Details on the methodology used can be found in the respective publications. We show the 95% confidence intervals (CI) when reported by the original study. *indicates that the estimated number of individuals was calculated here by multiplying the density reported in the study by the area of the respective protected area in https://ibapgbissau.org/areas‐protegidas/. ++Please note that the study by Dias et al. (2019) only considers 47km^2^ of study area. MI – indicates missing information in the original study.

In this study, we use geographically widespread genetic data and whole‐genome sequence data from multiple wild‐born individuals to estimate the *N*_*e*_ of the western chimpanzee population in GB. We aim to (i) estimate *N*_*e*_ and infer the recent and ancient demographic history of populations, (ii) integrate the different estimates to improve our understanding of both the evolutionary history and current demography of western chimpanzees, (iii) discuss the strengths and limitations of each *N*_*e*_ estimator method and their complementarity in informing conservation decisions for long‐lived organisms, and (iv) discuss the implications of the results for the conservation management of this emblematic species in GB.

## 2 Materials and Methods

### 2.1 Study Area

The study area covers a large proportion of the chimpanzee range in GB (Gippoliti and Dell'Omo 2003) (Figure 1b), encompassing an area of approximately 6,000 km^2^. Sampling of biological material was carried out in four geographically distinct and formally protected areas—1. Cantanhez National Park (CNP, 1,067.67 km^2^), 2. Cufada Lagoons Natural Park (CLNP, 890 km^2^), 3. Dulombi Natural Park (DNP, 1,600.96 km^2^), and 4. Boé National Park (BNP, 1,552.95 km^2^) (https://ibapgbissau.org/areas‐protegidas/). Chimpanzees were known to be present in these areas prior to our study (Gippoliti and Dell'Omo 2003).

### 2.2 Microsatellite Loci Dataset

We generated a dataset of 143 unique genotypes for 10 microsatellite loci derived from non‐invasive fecal samples (Borges 2017; Gerini 2018) (Figure 1b, Appendix S2). Eighty‐five genotypes correspond to samples collected between 2015 and 2017 in CLNP (*N* = 38), BNP (*N* = 34), and DNP (*N* = 13), and the remaining consisted of previously determined genotypes from CNP (*N* = 58) (Sá et al. 2013) (Figure 1b). Fecal samples were from unhabituated and unidentified individuals and preserved until DNA extraction following Ferreira da Silva et al. (2014). DNA extraction was carried out using two methods: (i) the QIAampDNA Stool Mini Kit (QIAGEN) at Michael W. Bruford research group laboratory facilities at the School of Biosciences, Cardiff University, UK (Sá et al. 2013), and (ii) the CTAB method (Vallet et al. 2008, adapted by Quéméré et al. 2010) for samples collected between 2015 and 2017, which were extracted at *Instituto Gulbenkian de Ciência* (IGC, Oeiras, Portugal) laboratory facilities. The procedures to avoid contamination by exogenous DNA are described elsewhere (Ferreira da Silva et al. 2014). DNA samples were identified to the species level using a mitochondrial DNA hypervariable region I fragment (as described in Sá et al. 2013, see details in Appendix S2). Allele size standardization between datasets was carried out using re‐extraction and re‐analysis of DNA extracts of five samples included in Sá et al. (2013) together with the novel samples analyzed in Borges (2017) and Gerini (2018). Allele scoring followed previously described procedures to guarantee minimal impact of allelic dropout and false allele errors and the presence of low‐quality genotypes (Appendix S2). The probability of identity (PI) and the probability of identity between siblings (PIsibs) (Waits et al. 2001), estimated using GenAIEx v.6.503 (Peakall and Smouse 2006), suggest that unique genotypes can be distinguished using six loci. Previous studies investigating population structure across southern GB, combining mtDNA, autosomal microsatellite markers, one Y‐linked microsatellite locus, Bayesian individual‐based clustering methods (i.e., STRUCTURE, BAPS), multivariate techniques (i.e., PCA, sPCA), analyses of molecular variance (AMOVA), pairwise *F*_ST_ values, and mtDNA haplotype networks, found no clear evidence of population structure or strong geographical patterns (Borges 2017; Gerini 2018). These findings suggested that until recently, chimpanzees exhibited considerable dispersal or had large populations across GB (Borges 2017; Gerini 2018).

### 2.3 Genomic Data

#### 2.3.1 Sampling

Whole‐genome sequences were produced from biological material collected from wild‐born chimpanzees: one roadkill (muscle sample T3‐Chimp collected in 2011 near CLNP) and four live individuals (blood samples from Bo, Bella, Simão, and Emília chimpanzees, collected between 2018 and 2019) confiscated by the Institute for Biodiversity and Protected Areas (IBAP) from private premises. We obtained the information that the individuals were caught in different sites within GB (e.g., Bo was originally from CNP, Bella was confiscated in Quebo but probably originated from CNP, Emília was from DNP, and Simão was living in Bafatá but traded in Quebo, Figure 1). Blood was collected as part of the placement of the individuals in a sanctuary abroad (Sweetwaters Chimpanzee Sanctuary, Ol Pejeta, Kenya, Ferreira da Silva and Regalla 2025, ^1^). The blood samples were drawn by a wildlife veterinarian (P. Melo, Vetnatura, https://www.vetnatura.pt/) for health screening and as part of a parasites and virus detection procedure before translocation (Melo et al. 2018). Samples were collected in 5 mL collection tubes filled with the anticoagulant ethylenediamine tetraacetic acid (EDTA) and preserved in cold until DNA extraction. The roadkill individual was found on the road next to CLNP (Figure 1b), and a muscle sample was collected and preserved in 98% ethanol until DNA extraction.

#### 2.3.2 DNA Extraction and Data Production

DNA was extracted from the five tissue samples adapting the method by Vallet et al. (2008). We used 500 μL of each blood sample and about 10 mg of muscle from the roadkill individual. The details of this two‐day DNA extraction protocol can be found in Appendix S3. We tested the quality of DNA extractions in 2% agarose gels and quantified DNA concentration using a Nanodrop microvolume spectrophotometer (ThermoFisher Scientific) (Table S4). Laboratory procedures took place at the IGC, and extractions were carried out in a biological safety cabinet in a Biosafety Level 2 dedicated room. Library preparation and sequencing were performed by Macrogen Inc. The sequencing library was prepared by random fragmentation of the DNA samples, followed by 5′ and 3′ adapter ligation or by “tagmentation”, which combines the fragmentation and ligation reactions into a single step. Sequencing libraries were prepared using the TruSeq Nano DNA library kit (350) and, subsequently, adapter‐ligated fragments were amplified by PCR and gel purified. The size of PCR enriched fragments was verified by checking the template size distribution by running an Agilent Technologies 2100 Bioanalyzer using a DNA 1000 chip. Libraries were sequenced using the Illumina HiSeq X and TruSeq platforms. Samples were sequenced in two events; the first one aimed at sequencing the muscle sample with a higher coverage of 30x and, after successful results, four blood samples were sequenced targeting a lower coverage of 15x.

#### 2.3.3 Whole‐Genome Sequence (WGS) Data Assembly, Mapping, and Genotype Calling

After all samples passed quality control tests, we used the BAM pipeline from PALEOMIX v. 1.3.2 to process the sequences for downstream analysis at the Globe Institute's (University of Copenhagen, Denmark) High‐Performance Computing (HPC) cluster. This pipeline trims adapter sequences, filters low‐quality reads, removes PCR duplicates, and aligns reads and maps them to a reference genome (Schubert et al. 2014); here we used the assembly Clint_PTRv2 (https://www.ebi.ac.uk/ena/browser/view/GCA_002880755.3) as the reference genome. During the adapter removal step, we discarded reads shorter than 25 bp after trimming. For the mapping, we used bwa‐mem and excluded reads with a mapping quality below 25. Finally, the PCR duplicates were identified and removed using the *MarkDuplicates* tool of the Picard toolkit (v. 2.6.0; https://broadinstitute.github.io/picard/).

For the genotype calling, we used the *HaplotypeCaller* algorithm in GATK (v. 4.2.0.0; Poplin et al. 2017) with the minimum base quality score set to 20. The GATK tool *SelectVariants* was used to obtain datasets containing only single nucleotide polymorphisms (SNPs). VCFtools (v. 0.1.16; Danecek et al. 2011) was used to remove sites with a quality score equal to or lower than 30, more than 10% of missing data, and mean depth values lower than 5 and higher than 100. A table describing the WGS data summary statistics for each sample, such as coverage, observed number of homozygous genotypes, expected number of homozygous genotypes, and inbreeding coefficient (*F*), can be found in Table S5.

As a quick assessment of the possible presence of genetic structure among the sampled individuals, we performed an analysis with the STRUCTURE software v. 2.3.4 (Pritchard et al. 2000) using the admixture model and assuming correlated allele frequencies (Silva 2024). The parameter set consisted of a burn‐in period of 50,000 steps, followed by 200,000 iterations, and 10 runs for each number of clusters (Van Wyngaarden et al. 2017; Silva 2024). The results indicated evidence for a single panmictic cluster (*K* = 1) as the best clustering solution to explain the observed genetic variation across individuals, that is absence of genetic structure (Silva 2024).

### 2.4 Effective Population Size Estimation and Demographic History

#### 2.4.1 The PSMC (Pairwise Sequentially Markovian Coalescent) and the IICR: Principles of Demographic Inference

The PSMC method of Li and Durbin (2011) was applied to the nuclear genomes of the five individuals for which tissue samples were obtained. The PSMC uses the information from the distribution of heterozygous sites along the genome of a single diploid individual (or two haploid genomes) and produces a curve where the *x*‐axis represents time, usually represented in a log scale, and the *y*‐axis is often interpreted as representing the effective population size. Proper scaling of the PSMC in years requires the use of estimates of generation time, mutation, and recombination rates.

PSMC v. 0.6.5‐r67 (Li and Durbin 2011) (available at http://github.com/lh3/psmc) was run on each individual genome using the following settings: −N25 −t15 −r5 −p “4 + 25*2 + 4 + 6”. Individual consensus sequences were generated using the *mpileup*, *bcftools*, and *vcfutils.pl* (*vcf2fq*) pipeline from SAMTOOLS v. 1.9, with minimum read depth (−d) set to five and maximum read depth (−D) set to thirty for individuals with coverage between 13 and 18 (samples: Bella_PT_GB, Bo_PT_GB, Emi_PT_GB, Simao_PT_GB, Table S5) and to 60 for the individual that had a coverage around 30 (sample T3‐Chimp, Table S5). This was done to follow a rule‐of‐thumb suggested by several authors (e.g., Hilgers et al. 2025, Cousins et al. 2025), in which the maximum read depth in the filtering is set to double of the average coverage of the data. The consensus sequence was converted into a fasta‐like format using the *fq2psmcfa* program, provided in the PSMC package, with the quality cut off (−*q*) set to 20. We assumed a mutation rate (*μ*) of 1.2 × 10^−8^ per base pair per generation and a generation time of 25 years (Besenbacher et al. 2019; Chintalapati and Moorjani 2020; Langergraber et al. 2012; Venn et al. 2014). To quantify the variance in PSMC curves, we performed 10 bootstraps per individual, following the re‐sampling protocol suggested by the authors. The inferred demographic histories for the five analyzed individuals were plotted in a single figure using Ghostscript 9.16 and Gnuplot 5.4.0. The PSMC plots are usually interpreted in terms of *N*_*e*_ changes but can also be interpreted in terms of connectivity changes (see Section 4).

#### 2.4.2 MSVAR Analysis of Microsatellite Loci Data

We used the Bayesian likelihood‐based approach of Storz and Beaumont (2002), as implemented in the MSVAR 1.3 software. This approach assumes a simple model of exponential population size change (allowing for either growth or decline) from an ancient population of size *N*_1_ to a present‐day population of size *N*_0_. In practice, the method uses a Monte Carlo Markov chain algorithm to estimate the posterior probability distribution of *N*_0_ and *N*_1_ and of the time at which the population started to increase or decrease (*T*, in years, assuming that a generation time is given), and the *per* locus mutation rate (μ). We conducted four independent runs with different initial values and varying sets of priors and hyperpriors to reflect assumptions of either constant population size (*N*_0_ = *N*_1_), population decline (*N*_0_ < *N*_1_), or population growth (*N*_0_ > *N*_1_) and therefore control for the impact of the priors on the posterior distributions (Table S6).

Analyses were run using a series of datasets to discard the possibility that the presence of related individuals, the sampling scheme, and undetected genetic structure, could impact the inferred demographic histories, and assuming a generational span of 25 years (following Langergraber et al. 2012). We aimed to recover the demographic history of western chimpanzees by analyzing samples from (i) GB as one population (*N* = 143 unique genotypes), (ii) by park (CNP *N* = 58, CLNP *N* = 38, DNP *N* = 13, and BNP *N* = 34 unique genotypes); (iii) a dataset formed by unrelated individuals (*N* = 121 unique genotypes, see below how relatedness was estimated); and (iv) five “random datasets” obtained by randomly selecting 58 unique genotypes, which correspond to the largest dataset from a single area (i.e., CNP). Datasets iii and iv were used to test the influence of the presence of highly related individuals and differences in sample sizes across datasets in demographic estimates, respectively. Analyses were also run for each park since coalescent theory predicts that when populations are structured, samples obtained from one population will tend to exhibit signals of bottlenecks, whereas samples obtained across many demes will tend to have a much weaker bottleneck signal (Beaumont 2004; Wakeley 1999), as shown in simulated data (Chikhi et al. 2010).

To estimate relatedness between individuals, we calculated the correlation coefficient between the observed and simulated values of relatedness (100 pairs) for the Milligan (2003) and Wang (2007) likelihood estimators using the *related* R package (Pew et al. 2015). We also estimated relatedness between pairs of individuals in the overall dataset and per protected area (CNP, CLNP, DNP, and BNP) using the ML‐Relate likelihood method (Kalinowski et al. 2006). We performed 100,000 simulations to identify dyads with a likely relationship of Parent‐Offspring or Full‐Siblings and *r* > 0.5 significantly different (*p* < 0.05) from dyads with a likely relationship of Half‐Siblings and Unrelated. For dyads identified using the full dataset and the park dataset, one genotype of the dyad, the one that displayed lower Quality Index (QI, Miquel et al. 2006), was removed from the dataset.

The individuals present in the five “random databases” were selected from the dataset (i) using the *runif* function in R.

Each run in MSVAR included 300,000 thinning update steps and 30,000 thinning intervals, totaling 9 × 10^9^ steps. We discarded the first 10% of each simulation to eliminate the influence of initial conditions on parameter estimation (burn‐in). We verified convergence between runs both visually and using Brooks, Gelman, and Rubin Convergence Diagnostic test (Gelman and Rubin 1992; Brooks and Gelman 1998) conducted in R version 2.11.1 (R Core Team 2010) using the package BOA version 1.1.7 (Smith 2007).

#### 2.4.3 Linkage Disequilibrium‐Based Estimation of Current *N*_*e*_ and Recent Demographic History From Genomic Data

We used GONE (Genetic Optimization for *N*_*e*_ Estimation) (https://github.com/esrud/GONE) that implements a genetic algorithm to infer the recent demographic history of a population from SNP data of one contemporary population sample (Santiago et al. 2020). The method can infer the demographic history of a population within the past few hundred generations, with the authors stressing that the greatest reliability and resolution is for the last 100 generations (Santiago et al. 2020). It uses the observed spectrum of linkage disequilibrium (LD) between pairs of loci over a wide range of genetic distances (implicit recombination rates) and has been validated by simulation under different demographic scenarios and for small sample sizes (i.e., *n* = 10, Santiago et al. 2020). These simulations suggested that not considering LD data between more distant loci (e.g., with scaled recombination rates > 0.05) in the analyses allows for better estimates of *N*_*e*_ trajectories, particularly when sample sizes are small or when there is population structure and migration rates between subpopulations are low (Santiago et al. 2020). Thus, the authors of the method recommend using a maximum recombination rate of 0.05, this being the default value of this parameter in GONE. Accordingly, we used this value and default settings for all other software parameters, such as no minimum allele frequency cutoff and 40 independent replicate runs, with the *N*_*e*_ point estimate for each generation being the geometric mean of the values of the replicates. Analyses were performed using 57,500 genome‐wide unphased autosomal SNPs without missing data. While ignoring pairs of loci with recombination rates greater than 0.05 helps in estimating demographic history trajectories with GONE, in theory using an upper bound of 0.5 for the recombination rate should be more adequate for estimating current *N*_*e*_ (Waples 2025). Thus, we also performed GONE analyses with this upper bound of 0.5 (with data analyzed for the last 100 generations and with 50 bins of pairs of SNPs). As suggested in Santiago et al. (2020), we obtained an empirical 95% confidence interval by running GONE on 20 random replicates of 57.5 K SNPs sampled from the whole‐genome sequences. Replicates were generated by variant thinning in PLINK 1.9 (www.cog‐genomics.org/plink/1.9/) (Chang et al. 2015).

Estimates in GONE can be affected by the presence of significant genetic structure (Santiago et al. 2020; Novo et al. 2023). But in this matter, the analysis we did in STRUCTURE showed no genetic structure among the five individuals and with no signs of external admixture (Silva 2024). Thus, we assumed all of the chimpanzees included in the GONE analyses belong to a single cluster.

#### 2.4.4 Linkage Disequilibrium Estimation of Contemporary *N*_*e*_ From Microsatellite Data

We used NeEstimator 2.1 (Do et al. 2014) to estimate contemporary *N*_*e*_ from microsatellite loci data using the bias‐corrected version of the method based on linkage disequilibrium (LD) (Waples 2006; Waples and Do 2010), which was originally developed by Hill (1981), and assuming random mating. The method is robust to equilibrium migration rates up to 10% at lower population sizes (Waples and England 2011; Gilbert and Whitlock 2015). We performed analyses both for the whole dataset and separately for each of the four protected areas. The software estimates confidence intervals (CIs), both parametric and based on jackknifing over individuals (Jones et al. 2016), which accounts for the fact that overlapping pairs of loci are being compared and implements a method to correct for possible biases due to missing data (Peel et al. 2013). In any case, for each of the five datasets, we also performed analyses removing loci with more than 10% missing data. When analyzing each dataset, and depending on its sample size, we used a minimum allele frequency (MAF), the Pcrit value, following the recommendations in Waples and Do (2010).

## 3 Results

### 3.1 Effective Population Size Estimation and Demographic History

#### 3.1.1 The PSMC (Pairwise Sequentially Markovian Coalescent) and the IICR

**FIGURE 2:**
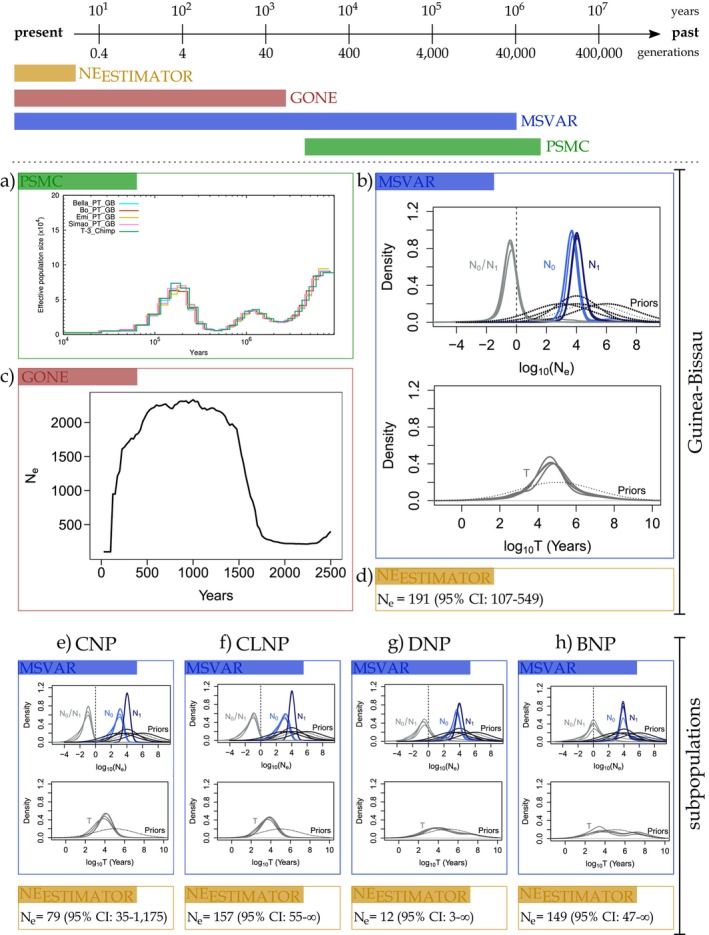
Graphical summary of results from four methods used to estimate the effective population size (*N*_*e*_) and infer the demographic history of the western chimpanzee ( *Pan troglodytes verus* ) in Guinea‐Bissau, shown at the national (a–d) and at the subpopulation/park scale (e–h, CNP, Cantanhez National Park; CLNP, Cufada Lagoons Natural Park, DNP, Dulombi National Park, and BNP, Boé National Park. Top panel: The four methods used in this study are positioned along the timeline according to their temporal resolution, that is the extent to which they allow for a robust estimate of the *N*_*e*_. The black arrow represents time, expressed in years (above arrow) and chimpanzee generations assuming a generation time of 25 years (below arrow). Central panel: At the Guinea‐Bissau scale (a‐d), we applied the four methods: two were based on whole‐genome sequence data from tissue samples (*N* = 5) — Pairwise Sequentially Markovian Coalescence (PSMC; a) and GONE (c)—and two were based on microsatellite loci from non‐invasive fecal samples (*N* = 143 unique genotypes)—MSVAR (b) and NeEstimator (d). (a) The trajectory of historical effective population size as inferred by PSMC. The *x*‐axis indicates time in years, assuming a mutation rate (*μ*) of 1.2 × 10–8 per base pair per generation and a generation time of 25 years, and on the *y*‐axis is indicated the effective population size scaled by the mutation rate (4*N*_*e*_μ) (× 10^4^). (b) The inference of the demographic history conducted using MSVAR v.1.3 for the whole dataset (*N* = 143 unique genotypes). Upper graph shows the posterior distributions of the contemporary effective population size (*N*_*0*_) and the ancestral population size (*N*_*1*_) for four independent runs. The bottom graph shows the posterior distribution for the time (in years) at which the demographic change has occurred (T), for four independent runs. Dashed lines represent prior distributions used for the three estimated parameters (Table S6). All values are represented on a log_10_ scale. (c) The recent demographic history inferred by GONE. The figure shows the results for the last 100 generations using five whole genome sequences. (d) Estimates of contemporary *N*_*e*_ and respective 95% confidence intervals, obtained with NeEstimator from the microsatellite data for the whole dataset. Bottom panel: The results of MSVAR and NeEstimator analyses using the microsatellite loci data for the parks datasets (e) CNP, *N* = 58; f) CLNP, *N* = 38; g) DNP, *N* = 13 and h) BNP, *N* = 34 unique genotypes).

The PSMC curves exhibited a series of increases and decreases of *N*_*e*_, with the last decrease starting around 200 kya (Figure 2a). The results for the 10 bootstrap replicates for each individual were highly consistent (Figure S7a–e).

#### 3.1.2 MSVAR Analysis of Microsatellite Data

The analyses conducted with the control datasets (unrelated and “random datasets”) did not provide significantly different results from the demographic scenarios obtained for the complete datasets of GB and the parks, hence suggesting that relatedness and sample size did not significantly affect the results.

From the whole dataset (*N* = 143 unique genotypes), MSVAR estimated a contemporary *N*_*e*_ (*N*_o_) between 4500 and 6500 breeding individuals, which resulted from a mild bottleneck starting about 44,500–70,000 years ago from a more ancient *N*_*e*_ around 10,000–12,000 (*N*_1_) (Figure 2b). To account for the possible effects of genetic structure, we also carried out analyses separately for each park (Figure 2e–h). For the CNP chimpanzee population, MSVAR identified a stronger signal with estimates of *N*_*0*_ between approximately 500 and 1125 breeding individuals, whereas *N*_1_ estimates were between 10,000 and 12,000 breeding individuals, with limited overlap between the *N*_0_ and *N*_1_ median posterior distributions and a posterior distribution of *N*_0_/*N*_1_ that was consistently below zero (Figure 2e, Table S7). MSVAR estimated that this demographic decrease of the CNP chimpanzee population occurred around 5000 and 12,500 years ago under the assumption of panmixia (Table 2).

**TABLE 2:** Estimation of long‐term effective population size (*N*_*e*_) of the western chimpanzee ( *Pan troglodytes verus* ) in Guinea‐Bissau, for the whole dataset and per park using microsatellite loci data and employing the Bayesian likelihood‐based approach implemented in MSVAR 1.3 (Storz and Beaumont 2002).

Site	*N*	*N*_*0*_, contemporary (breeding individuals)	*N*_*1*_, ancient (breeding individuals)	Time ago of demographic change (years)	Estimated demographic trajectory
Whole dataset	143	4,411–6,510	10,705–11,910	44,596–69,518	Evidence of mild demographic bottleneck
Cantanhez National Park	58	566–1,125	10,615–11,644	5,288–12,477	One order of magnitude demographic bottleneck
Cufada Lagoons Natural Park	38	534–1,225	10,397–11,416	3,612–7,874	One order of magnitude demographic bottleneck
Dulombi National Park	13	2,769–7,401	9,356–10,651	13,110–32,085	Stable
Boé National Park	34	6,716–24,642	7,320–8,555	6,753–85,645	Stable

*Note:* In the table is the number of genotypes (*N*), the posterior distributions of contemporary effective population size *N*_0_ (breeding individuals), the ancient population size *N*_1_ (breeding individuals), the time at which the population started to change (in years, assuming a 25 years generation time) for four independent runs for each dataset considered and the estimated demographic trajectory. The range between the lowest and highest median across the four independent runs is indicated for *N*_0_, *N*_1_ and T.

We found a similar demographic scenario for the CLNP chimpanzee population (Figure 2f), which is also located in the coastal area and is geographically close to CNP (Figure 1). The CLNP chimpanzee population was also found to have undergone a one‐order‐of‐magnitude bottleneck, with very similar values of *N*_0_ and *N*_1_ to the inferred scenario at CNP (Table 2). The putative difference between the demographic histories of the two coastal populations is the fact that the demographic decrease may have occurred later in CLNP than in CNP (CLNP: 3,612–7,874 years ago), but the inferred posterior distributions of T for both parks still overlap considerably (Table 2, Figure 2e,f). The bottleneck signal of both populations is also confirmed by the *N*_*0*_ */N*_*1*_, which in both cases is below 0, across the four simulated scenarios (Figure 2e,f).

As for the chimpanzee populations from inland, at DNP and BNP (Figure 1), we found different demographic histories (Figure 2g,h). Not only are the current *N*_*e*_ much larger but there is also no clear signal of demographic change for any of these regions. The population from DNP is estimated to have a *N*_0_ between 2,769 and 7,401 breeding individuals, with a slightly larger estimated *N*_1_ (Table 2). However, the variation in *N*_*0*_ estimates across the four different simulated scenarios does not allow for a clear signal of population size change. This absence of a clear bottleneck signal is also supported by the variation of the *N*_0_/*N*_1_ estimates and their overlap with zero (meaning no population size change), which is also translated into weak convergence of the *T* posterior distributions (Figure 2g). For the easternmost chimpanzee population in GB sampled in BNP (Figure 1), the inferred posterior distributions of *N*_*0*_ and *N*_*1*_ are very consistent between the different runs, which indicates a large and historically stable population in this park (Figure 2h). The estimated values for the *N*_0_ indicate a population with a current effective population size above 6500 breeding individuals (Table 2). The estimated posterior distributions of *N*_1_ extensively overlap with the *N*_0_ estimates, as it is also clear from Figure 2h suggesting no population size changes. The scenario of a large and historically stable population is further supported by the *N*_0_/*N*_1_ estimates consistently overlapping with zero (Figure 2h).

Please note that one has to be careful in interpreting the MSVAR results as the *N*_0_ and *N*_1_ values could also be interpreted in models of population structure with or without population size change, and where *N*_0_ could correspond to the current size of the local demes, and *N*_1_ to the effective size of the ancient metapopulation. This is addressed in the Section 4.

#### 3.1.3 Linkage Disequilibrium‐Based Estimation of Current *N*_*e*_ and Recent Demographic History From Genomic Data

Analyses in GONE with default settings (i.e., considering only pairs of loci with a recombination rate of up to 0.05) gave a mean current *N*_*e*_ estimate of 100 breeding individuals (95% CI: 89–111). In terms of demographic history over the last 100 generations, GONE estimated a tenfold growth of *N*_*e*_ from about 220 approximately 80 generations ago, to a plateau at 2,100–2,200 breeding individuals, 20–50 generations ago, followed by a steady decline to the present (Figure 2c). Please note that the tenfold increase 70–60 generations ago may be an artifact, and analyses with larger sample sizes are needed to verify this result. Similarly, although a decline of *N*_*e*_ in GB chimpanzees in recent generations could be expected, this decline needs to be corroborated by analysis of larger samples; moreover, this decline may also reflect a transition, going backwards in time, from a local *N*_*e*_ to a *N*_*e*_ relative to a larger spatial/temporal scale (e.g., ancestral metapopulation) (Mazet et al. 2016; Fedorca et al. 2024; Waples 2025). In the analyses using all recombination bins (i.e., hc = 0.5), which should provide better estimates of contemporary *N*_*e*_, the mean estimate of current *N*_*e*_ was 489 breeding individuals (95% CI: 395–583).

#### 3.1.4 Linkage Disequilibrium Estimation of Contemporary *N*_*e*_ From Microsatellite Data

Estimates of contemporary *N*_*e*_ and respective 95% confidence intervals, obtained with NeEstimator from the microsatellite data, both for the whole dataset and separately for each of the four parks, are presented in Table 3. The *N*_*e*_ point estimates for the whole dataset were around 150–190 breeding individuals, with the 95% CIs spreading around 80–550 breeding individuals. Point estimates for CLNP and BNP were of similar magnitude, respectively at about 160–230 and 130–150 breeding individuals, but the 95% CIs had infinite upper bounds (Table 3). The *N*_*e*_ point estimates for the CNP (54–79 breeding individuals) were substantially lower, although their 95% CIs overlapped with those of populations from the other parks (Table 3). On the other hand, the point estimates for DNP, at 8–12 breeding individuals, were approximately an order of magnitude smaller than for the other datasets and with parametric 95% CIs not overlapping with the other parks; yet, the jackknife CIs had infinite upper bounds (Table 3).

**TABLE 3:** The effective population size (*N*_*e*_) estimates and jackknife‐on‐samples and parametric 95% confidence intervals of the western chimpanzee ( *Pan troglodytes verus* ) in Guinea‐Bissau obtained with neestimator from microsatellite data.

	*N*_*e*_	95% CI (JackKnife)	95% CI (Parametric)
Whole dataset (*N* = 143; *P*_*crit*_ = 0.02)			
10 loci	191	[107, 549]	[138, 294]
6 loci	146	[82, 376]	[98, 252]
Cantanhez National Park (*N* = 58; *P*_*crit*_ = 0.02)			
10 loci	79	[35, 1,175]	[52, 143]
6 loci	54	[26, 215]	[34, 103]
Cufada Lagoons Natural Park (*N* = 38; *P*_*crit*_ = 0.02)			
10 loci	157	[55, ∞]	[70, ∞]
9 loci	229	[65, ∞]	[79, ∞]
Dulombi National Park (*N* = 13; *P*_*crit*_ = 0.05)			
10 loci	12	[3, ∞]	[6, 30]
7 loci	8	[2, ∞]	[3, 23]
Boé National Park (*N* = 34; *P*_*crit*_ = 0.02)			
10 loci	149	[47, ∞]	[60, ∞]
7 loci	133	[32, ∞]	[50, ∞]

*Note:* The results shown are for the whole dataset and for each park separately. Sample size (*N*, unique genotypes) and *P*_*crit*_ (minimum allele frequency cut‐off) used for each dataset are indicated. The choice of *P*_*crit*_ followed the recommendations of Waples and Do (2010). For each dataset, we ran analyses using all ten genotyped loci and using only loci with less than 10% missing data. *N*_*e*_ values were rounded to integers.

## 4 Discussion

In this work, we used several methods that aim to estimate either a single effective population size or possible changes in *N*_*e*_ over different temporal scales, using samples obtained over different spatial scales. We estimated the demographic trajectories of western chimpanzees representative of the whole country and, separately, for four geographic populations inhabiting parks in the south of the country, which are considered relevant areas given the global conservation of the subspecies.

### 4.1 Estimation of *N*_*e*_ Over Different Temporal Scales

As noted in Luikart et al. (2010) review and in Box 1 in Ryman et al. (2019), one can consider, as a simple approximation, the idea that different *N*_*e*_ estimators should be interpreted by using different time frames (Luikart et al. 2010; Wang 2005). Some estimates would correspond to the “ancient time” (from hundreds to tens of thousands of generations), whereas others would correspond to the recent or contemporary *N*_*e*_ (from a few to tens or hundreds of generations). Typically, MSVAR (microsatellite data) and PSMC (single genome data) provide information about the former time period, whereas NeEstimator (microsatellite data) and GONE (WGS data) provide estimates that are mainly about the recent past (Figure 2). The latter type of methods assumes that the long‐term *N*_*e*_ can be to some extent neglected regarding some properties of the genetic data, such as the LD pattern (at least among some markers) or the variation of allele frequencies in the last few generations. We note that MSVAR also provides an estimate of contemporary *N*_*e*_ but as part of a demographic model of size change, and that GONE also integrates the contemporary *N*_*e*_ in a trajectory of *N*_*e*_ change. The contemporary *N*_*e*_ estimates are considered the most relevant to assess extinction risk because they reflect ongoing or recent demographic or reproductive processes, whereas the historical *N*_*e*_ refers to the genetic or demographic processes over much longer periods, which can elucidate how the population dynamics were affected by past environmental changes (Luikart et al. 2010; Santos‐del‐Blanco et al. 2022). We argue that effective conservation decisions should be guided by the integration of these various estimates (Figure 2), while also considering the specific strengths and limitations of each method.

The PSMC method has been used on many endangered species since it only requires one genome sequence and is thus adapted for endangered species for which large genomic data sets are difficult to obtain. However, its interpretation is usually done in terms of changes in *N*_*e*_ only, and we stress here that the demographic history should be assessed with multiple methods, suitable for different spatial and temporal scales, and more realistic models that allow for different scenarios of past population structure (Carbone et al. 2014; Teixeira et al. 2021; Guevara et al. 2021). Implicitly, what the PSMC recovers is the distribution of coalescence times along the genome, and more work is needed to clarify the recent evolutionary history of these species. Furthermore, in the last 25 years, there has been an increasing recognition that population structure can generate spurious signatures of population size change (Beaumont 2004; Chikhi et al. 2010; Wakeley 1999). In the specific case of the PSMC method, Mazet et al. (2016) showed that it is in fact impossible to determine whether a particular PSMC plot is the result of real change in *N*_*e*_ or of a more complex model of population structure with changes in connectivity, without any change in population size. In the latter case, it thus becomes impossible to make statements regarding changes in *N*_*e*_ from changes observed in the PSMC curve alone. Mazet et al. (2016) introduced the concept of IICR (inverse instantaneous coalescence rate) and noted that the PSMC method in fact infers the IICR, not *N*_*e*_. The IICR will be identical to *N*_*e*_ under panmictic models without population structure but very different from any *N*_*e*_ changes as soon as there is population structure. Altogether, this suggests that for species like chimpanzees that are known to be structured (Fünfstück et al. 2015; Lester et al. 2021), signals of population size changes inferred from methods assuming panmixia (PSMC, MSVAR, Bottleneck, StairwayPlot, GONE, etc.) must be interpreted with caution (Steux et al. 2024).

The PSMC plots that we obtained with the five individuals from GB exhibited a similar trajectory to those obtained by Prado‐Martinez et al. (2013), which compared the genetic diversity and demographic history of all great apes, including western chimpanzees but from different locations. The authors estimated the peak of effective population size for the western chimpanzee at ~150 kya, which is similar to the time of the highest *N*_*e*_ estimated here (around 200 kya). The difference in these values could be due to the fact that we used the chimpanzee reference genome, whereas Prado‐Martinez et al. (2013) used the human genome as a reference. However, these differences are minimal, probably because the divergence between *Homo sapiens* and *P. troglodytes* is on the order of 1% (see Prasad et al. 2022 for the effect of divergence of the reference genome).

Beyond these important technical issues, the PSMC curves obtained can be interpreted in terms of changes in *N*_*e*_, assuming panmixia and no population structure, in terms of changes in connectivity under population structure (Steux et al. 2024) and constant size or as a combination of both types of changes. Altogether, changes in *N*_*e*_ suggest that the populations sampled were part of a metapopulation that may have been very large in the past and have been significantly reducing for the last 200 kya or are the result of a metapopulation that was characterized by changes in connectivity with no obvious population decrease during that period or, more likely, a combination of both changes in *N*_*e*_ and connectivity. However, whether one considers population structure or panmixia, our results suggest that the chimpanzee populations sampled were part of a metapopulation that may have been very large and included all the regions sampled in this paper. We will come back to this later.

The analyses using the MSVAR method and microsatellite loci data suggested that chimpanzees from GB have undergone a mild demographic decrease starting around 40,000 years ago (considering the whole dataset). However, for the analyses of the different parks, we inferred contrasting histories for inland (eastern areas, i.e., DNP and BNP) and coastal (western areas, i.e., CLNP and CNP) populations, with either no changes or recent and minor *N*_*e*_ changes. Also, the estimates for *N*_*1*_ were very similar for the different parks with ~10,000 breeding individuals. This may indicate that the populations at the four parks were connected in the past and were part of one metapopulation (itself probably connected to other regions outside GB). This result is in agreement with our interpretation of the PSMC curves. By contrast, the estimates of *N*_*0*_ by MSVAR were much smaller, indicating either that population connectivity changed towards more recent times, or that these values correspond to the local deme size, as expected under the coalescent theory of structured models. Whichever interpretation one may favor, our results appear to suggest that, whereas the inland eastern populations remained large/connected with other regions, the western coastal populations appear more isolated, a process that may have started thousands of years ago at the scale of the country. We have to be cautious with these figures as the timing of size change inferred by MSVAR may not correspond to any particular timing of change in gene flow, since models without changes in connectivity would also generate signals of bottleneck. Despite these cautionary remarks, we believe that the CNP and CLNP may have become separated from the metapopulation, but could have remained connected, forming a smaller sub‐population not exceeding 1,200 breeding individuals. This result also suggests that chimpanzees may have still been able to disperse between these two parks in the last generations. On the other hand, DNP and BNP may be transnational populations, given the geographic proximity to the Republic of Guinea (Figure 1).

In a recent study, Fontsere et al. (2022) analyzed chromosome 21 genome‐wide data across the chimpanzee species and subspecies distribution and inferred a large exchange of migrants during the last ~800 years (range 117–2,200 years) for the populations located in the northern range of the distribution, which includes Senegal, Mali, northern Guinea, and GB. The authors used samples from BNP only (Fontsere et al. 2022). Thus, no signs of long‐term isolation were detected for GB (Fontsere et al. 2022). In another study, Heinicke, Boesch, et al. (2019) investigated the existence of subpopulations across the *P. t. verus* distribution based on field survey data and spatial modeling tools. According to these authors, one large subpopulation (> 33,000 individuals, representing approximately 50% of the total population size) was predicted at the northern range of the subspecies, in areas characterized by savanna‐mosaic habitats and extending across the Fouta Djallon highland region and the neighboring areas of Senegal and GB (including the four parks of our study), up to Mali and Sierra Leone (Figure 1). Thus, these studies could explain why we inferred large *N*_1_ estimates of ~10,000 breeding individuals (compared to *N*_0_). These estimates could correspond to the whole GB population, possibly reflecting a historical connection of the GB population to a large metapopulation centered in the Fouta Djallon highland region.

In a more recent study, Steux et al. (2024) suggested that patterns of genomic variation as observed in the PSMC curves could be modeled as part of a metapopulation of small demes characterized by periods of changing connectivity. They estimated that, in comparison to other *P. troglodytes* subspecies, the *P. t. verus* population was characterized by smaller demes, which could explain the lower nucleotide diversity observed in western chimpanzees (Steux et al. 2024). Their study, however, focused on the rather ancient past (older than ca. 50 kya) due to the uncertainties on the PSMC curves they analyzed in the recent past (Steux et al. 2024). Other genetic studies based on microsatellite loci and a fragment of the mitochondrial DNA D‐loop region using data from GB populations do not indicate a strong population structure (Borges 2017; Gerini 2018; Sá et al. 2013), which suggests that the chimpanzees can disperse between parks. The change in connectivity between inland (DNP‐BNP) and coastal areas (CNP‐CLNP) within GB cannot be directly inferred from our analyses when interpreting results. However, our analyses do identify a large ancestral population that might have been fragmented possibly as a consequence of environmental changes that occurred around 10,000 years ago. This may include climate instability in West Africa (see below) and the more recent increased anthropogenic impact resulting from the development of agriculture. The Younger‐Dryas Holocene transition between the African Humid Period (14,500–6,000 years ago), characterized by the expansion of forests and lakes across the Sahara region, and a sequence of time periods characterized by arid conditions towards the late Holocene (Gasse and Van Campo 1994), was very abrupt. This period of climate instability, with a succession of warm and cooling events in West Africa, impacted the extension of forest cover (De Menocal et al. 2000) and, most likely, the size and connectivity of the populations of forest‐dwelling fauna, as is the case of the western chimpanzee.

The GONE analyses suggested a somewhat surprising growth of *N*_*e*_ between 1750 and 1250 years ago (i.e., in generations 70–50) followed by a roughly stationary period until 500 years ago (i.e., between generations 50 and 20) and then a relatively rapid massive decrease until today. This pattern, in particular the substantial increase 70–50 generations ago, may be an artifact of the method due to the small number of individuals included in the analysis (e.g., five chimpanzees). For example, Beichman et al. (2018) illustrated the general difficulties of demographic history methods in inferring demographic events over the past hundred generations using whole‐genome data for fewer than 10 individuals. Reid and Pinsky (2022) also emphasized the importance of large sample sizes for different demographic history methods regarding power and precision to detect and quantify population declines in the last 100 generations, with GONE being no exception to a sharp deterioration in performance when sample sizes are small. Similar and even larger oscillations in *N*_*e*_ have also been described by Santiago et al. (2020) when they simulated data from a simple structured model (panel F of their Figure 2), but at this stage one should be cautious to interpret these results until larger sample sizes are analyzed. The rapid and substantial decline in *N*_*e*_ over the last 20 generations suggested by GONE can be seen as plausible, and in agreement with the results of other methods used in this study (e.g., the NeEstimator estimates of current *N*_*e*_ using the microsatellite data), but this trajectory could also be the result, going backwards in time, of a transition from a local *N*_*e*_ to a *N*_*e*_ referring to a (much) larger spatial and temporal scale, and thus also reflecting the signal of the ancestral metapopulation (Fedorca et al. 2024).

The *N*_*e*_ point estimates for the whole dataset obtained with NeEstimator were around 150–190 breeding individuals (95% CIs spreading around 80–550). The fact that the *N*_*e*_ point estimate for the whole dataset (‘metapopulation’) is of the same order of magnitude as the point estimates for some of the individual parks suggests that the former may be an underestimate. It is well known that the LD method can underestimate *N*_*e*_ in the presence of marked genetic structure (Neel et al. 2013; Gilbert and Whitlock 2015; Mergeay et al. 2024). Past studies using genetic data from chimpanzee populations in GB did not find strong population structure (Borges 2017; Gerini 2018; Sá et al. 2013), but most individuals in the country may currently inhabit separated parks (e.g., an estimated 65% of chimpanzees, Heinicke, Boesch, et al. 2019), and thus connectivity between parks may have been reduced during the last decades. Nevertheless, within each protected area, samples were collected randomly from unidentified individuals in a scheme compatible with the assumption of random mating.

The 95% confidence intervals of *N*_*e*_ point estimates for CLNP (160–230 breeding individuals) and BNP (130–150 breeding individuals) had infinite upper bounds. This can happen if *N*_*e*_ is large and/or if the data have limited information (e.g., insufficient sample size; reduced number or not very polymorphic loci) (Marandel et al. 2019). The infinite upper bound implies that it is not possible to reject the null hypothesis that LD can be explained entirely by sampling error (Waples and Do 2010). Still, the finite lower bound provides useful information about the minimum limit of *N*_*e*_ (Waples and Do 2010). For DNP (*N*_*e*_ estimated at 8–12 breeding individuals, finite parametric 95% CIs not overlapping with the other parks), only the jackknife CIs had infinite upper bounds. The small sample size from the DNP (*N* = 13 unique genotypes) may contribute to an underestimation of *N*_*e*_. This underestimation will tend to be smaller if the true *N*_*e*_ is not very large (e.g., ≤ 100) and will be greater the larger the true *N*_*e*_ (Waples and Do 2010). A correlation between sample size and estimated *N*_*e*_ has been observed previously for the LD method, but also for other methods for estimating current *N*_*e*_ (Skrbinšek et al. 2012; Kimble et al. 2023; Cox et al. 2024).

### 4.2 Implications for Conservation Management of the Western Chimpanzee in Guinea Bissau

Franklin et al. (1980) proposed the thresholds of 50/500 for a minimum effective size required for a viable population in the short and long term, respectively. This recommendation became an established rule of thumb in conservation biology and has been proposed as a genetic indicator to assess progress towards global conservation targets (Frankham et al. 2014; Hoban et al. 2020). The 50 short‐term rule refers to an effective size quantifying the rate of inbreeding (*N*_*eI*_). The minimum *N*_*eI*_ of 50 individuals is thought to be enough to prevent the rapid inbreeding of the population (i.e., 1% per generation), which could lead to excessive homozygosity for deleterious recessive alleles and reduced fitness by inbreeding depression. The 500 long‐term rule refers to the effective size related to the loss of additive genetic variation (*N*_*eAV*_). This threshold defines the *N*_*e*_ above which a population should retain enough evolutionary potential to adapt to new selective forces (i.e., future environmental conditions, Jamieson and Allendorf 2012). Subsequently, these numbers have been doubled, with 100 individuals being presented as more adequate to prevent inbreeding depression over five generations for wild populations (i.e., limiting to 10% the loss in total fitness) and 1000 individuals as necessary to protect evolutionary potential in the long term (Frankham et al. 2014), particularly when a species' reproductive rate is low (Pérez‐Pereira et al. 2022). When a population is detected to have a small or declining *N*_*e*_, managers and conservationists should be called to investigate the most likely causes and to reverse the demographic trajectory (Wang et al. 2016). This is why estimating *N*_*e*_ is increasingly recognized as central to conservation programs.

While estimates from different methods may vary, results consistently suggest that the current *N*_*e*_ for each park is below 500 breeding individuals. However, considering that functional connectivity might still be maintained across some of these parks, particularly within coastal and inland populations as our results suggest, the current *N*_*e*_ for the overall GB chimpanzee population should exceed the 500‐individual threshold. This implies it may still retain the evolutionary potential of the metapopulation.

This seemingly promising result does not imply that the GB chimpanzee population is without conservation concerns. On the contrary, it indicates that the long‐term viability of the populations from each park is highly dependent on gene flow from other populations. If further habitat changes lead to decreased population functional connectivity, inbreeding depression can pose a serious threat to the chimpanzees in GB. While no *N*_*e*_ estimate should be taken at face value, the fact that all estimates were qualitatively similar when using different methods, making different assumptions, and using different types of genetic markers suggests that the contemporary demographic dynamics of chimpanzees in GB are driven by already small and isolated coastal populations and still larger and possibly connected inland populations, with all demes deriving from what used to be a very large metapopulation. However, it is important to note that even estimates of contemporary *N*_*e*_ refer to the parental population of the sampled individuals, meaning that these estimates may reflect the effective size of the population a few generations back (25–100 years ago). Furthermore, these results confirm the selection of the coastal areas of GB and the Republic of Guinea by Kormos and Boesch (2003) as priority regions for conservation of the subspecies and are aligned with estimates of density that point to a small population in CLNP (Carvalho et al. 2013; Table 1), highlighting the critical conservation situation of these populations.

Small populations (i.e., < 500 breeding individuals) may go extinct through a phenomenon referred to as extinction vortex (Gilpin and Soulé 1986), in which genetic and demographic issues interact synergistically, decreasing genetic diversity and mean fitness, resulting in a lower population growth rate. This results in further decreases in genetic diversity and promotes the subsequent processes to happen in a cascade until the extinction of the population. In the case of the western chimpanzees in GB, the main conservation threats have been identified and characterized to some extent. Natural habitats have generally been converted into subsistence crop plantations, such as rice (*Oryza* spp.) or cassava ( *Manihot esculenta* ), and cashew ( *Anacardium occidentale* ) monoculture agroforests at least for the past two decades (Hockings and Sousa 2013, Temudo and Abrantes 2014). Additionally, the construction of roads and other infrastructures increased the accessibility to remote areas and promoted more encounters with humans, which may have increased chimpanzee mortality. Although it was found that chimpanzees can cross and use human‐altered habitats to some degree, namely sharing the use of forested and village areas with local communities (e.g., in CNP, Bersacola et al. 2021), extensive habitat loss and conversion into crop fields and villages are expected to reduce connectivity between populations and diminish population size rapidly (Torres et al. 2010). In CNP, for instance, it was estimated a loss of 11% of suitable habitat and the death of between 157 and 1103 individuals in the population for the period between 1986 and 2003 (Torres et al. 2010), a time period that corresponds to less than one chimpanzee generation. Moreover, as reported by Stiles (2023) and as illustrated here (with the four blood samples obtained from confiscated individuals), hunting for live individuals to supply the national and international illegal pet trade occurs in the country (Ferreira da Silva and Regalla 2025; Stiles 2023). Quantitative data on the number of traded chimpanzees originating from the GB in international trade routes is missing (Clough and Channing 2018; Ferreira da Silva and Regalla 2025; Stiles et al. 2013; Stiles 2023). However, given the ease of detecting chimpanzees in private houses and hotels (Ferreira da Silva and Regalla 2025) and considering that five to 10 adults can be killed to harvest one single infant chimpanzee (Teleki 1980), it can be suggested that hunting to supply the trade of live individuals may have contributed to a reduction of the population and consequently of the *N*_*e*_. Furthermore, as conservation threats tend to act synergistically at the local and regional scale—habitat fragmentation increases accessibility to natural habitats, which in turn, increases poaching, negative interactions with farmers, and disease transmission (Humle et al. 2016)—the negative impacts of human‐derived activities on the population effective size may be larger than each threat is individually.

Chimpanzees inhabiting CNP and CLNP—the two populations identified in this study at a high risk of extinction—are currently negatively impacted by habitat loss and fragmentation and by retaliatory killing by farmers during crop‐foraging (Hockings and Sousa 2013). Chimpanzees in CNP may also be subjected to higher reproductive isolation since the park is on a peninsula surrounded by two permanent water bodies that are insurmountable by chimpanzees, and suitable habitat for the subspecies was considerably lost in northwestern areas where the isthmus connects the peninsula to the mainland (i.e., Bantael Sila, Cumbijã, and Guiledge villages, Torres et al. 2010). Furthermore, these coastal areas have been considered important to maintain gene flow with the GB mainland for another primate species (e.g., Guinea baboons, *Papio papio* , Ferreira da Silva et al. 2014; Ferreira da Silva et al. 2018). Similarly, other forest‐dwelling and hunted primate species at CNP may have gone through a *N*_*e*_ decline of a like magnitude. Minhós et al. (2016) found a pattern of *N*_*e*_ decrease for co‐distributed populations of colobus monkeys inhabiting CNP ( *Piliocolobus badius temminckii* and *Colobus polykomos* ) at a similar time period as estimated here for CNP chimpanzees (i.e., ca. 10,000–3,000 years ago). The *N*_*e*_ for colobus monkeys was estimated using alike models and genetic markers (i.e., MSVAR runs and microsatellite loci, in Minhós et al. 2016). The fact that a similar demographic event was observed for other co‐distributed species in CNP, despite different socio‐ecological features, strengthens our interpretation that CNP chimpanzees experienced low *N*_*e*_ in recent times. Moreover, several chimpanzee communities across CNP show signs of leprosy (caused by *Mycobacterium leprae* , Hockings et al. 2021), which is likely, but so far unknown, to have negative impacts on the longevity and reproductive success of individuals. On the other hand, in the southern region of the other coastal park—CLNP, the construction of a large road and a thermoelectric plant with respective electricity transmission lines led to the loss of one of the best‐preserved forest patches of the park (Catarino 2019) and increased the accessibility to areas previously identified to be used by chimpanzees for nesting (Carvalho et al. 2013). Cases of live chimpanzee captures have been recorded in both parks, which most likely implies adult mortality (Ferreira da Silva and Regalla 2025; Ferreira da Silva, Minhós, et al. 2021). Furthermore, CLNP is bordered in the east by the main road connecting the south of the country to the capital city—Bissau and, as demonstrated in this study, wildlife‐vehicle collisions do happen. Our results suggest that CLNP and CNP chimpanzee populations are at high risk of extinction and that human‐derived activities potentially threatening these individuals should be investigated.

Our study provides the first estimates of *N*_*e*_ for DNP. The census size of this subpopulation has not been estimated by past studies. We estimated a historical *N*_*e*_ of 2,769–7,401 breeding individuals using MSVAR but could not detect a strong departure from mutation‐drift equilibrium. This result was at odds with the very low contemporary *N*_*e*_ of 6–30 breeding individuals obtained using NeEstimator. DNP is located at the northern margin of the Corubal River and is currently at one edge of the subspecies distribution (Figure 1). Our estimates of a stable demographic trajectory and a large historical *N*_*e*_ suggest that DNP was at some point in time connected to other large chimpanzee populations. Connectivity to populations located in the south of the Corubal River should be reduced at present times because the current width of the water body, which can surpass one kilometer in some sites, may be a significant barrier to primates' gene flow. This was found for other GB primates, namely the green monkey ( *Chlorocebus sabaeus* , Colmonero Costeira et al. 2025). Nevertheless, the configuration and discharge of the Corubal River may have been different in the past. The mouth of the Corubal River may have been located more to the southwest part of the country, surrounding the current location of the *Rio Grande de Buba* (Buba channel) area (Alves 2007), which could have allowed chimpanzees to cross the watercourse. The small contemporary *N*_*e*_ found for the DNP chimpanzee population may also be explained by the fact that present environmental conditions do not support a large population of chimpanzees. DNP is located at the edge of the distribution of the subspecies (Figure 1) and has a low density of villages and other human infrastructures. This area is mostly dominated by woodlands and savannah woodland formations (Catarino et al. 2008) and was found to be of low habitat suitability for chimpanzees by modeling exercises (< 100, range 0–1,000, in figure S2.2 in Carvalho et al. 2021), which could be either related to environmental conditions or a small sample size (J. Carvalho, personal communication). During fieldwork at DNP, chimpanzees were mostly detected (and fecal samples collected) in greater proximity to gallery forests along smaller streams or next to the Corubal River (Figure 1b), and we observed that the subspecies was not widely distributed in the park area, as it is in CNP, for instance (Sá et al. 2013). Chimpanzees at Fongoli in Senegal, inhabiting a similar open savanna‐woodland environment to the DNP chimpanzee population, do not suffer from nutritional stress but display physiological stress from dehydration and heat, which does not seem to be behaviorally compensated by sitting for longer periods in the shade or using pools or caves, for instance (Wessling et al. 2018). Such adverse environmental conditions may be determinant for constraining the distribution at the biogeographical range limits of the subspecies (Wessling et al. 2018) and similarly limiting the size of the population at DNP in GB.

By contrast, our estimates of historical *N*_*e*_ of the population of chimpanzees inhabiting the BNP (MSVAR *N*_*0*_: 6,716–24,642 breeding individuals) are large and confirm the classification of the area as stable or of high density (Heinicke, Mundry, et al. 2019). The Boé population has been included in a previous population genomic study, using samples collected across the subspecies range (Fontsere et al. 2022), which estimated high and recent connectivity (for the last ~780 years, range 117–2,200 years) between communities at the northern range (localities in the Republic of Guinea and south of Senegal, figure 3 in Fontsere et al. 2022). The Boé chimpanzees were found to be genetically closer to the ones in southern Senegal (Fontsere et al. 2022), possibly due to long‐term connectivity between the two neighboring populations. Present‐day high density of chimpanzees in the Boé region has been justified by (i) remoteness of the area and difficult access, (ii) rare hunting of chimpanzees to comply with religious taboos, (iii) high habitat suitability for chimpanzees, and (iv) slow habitat loss and conversion, and in a large area, habitats are undisturbed (Binczik et al. 2017; Carvalho et al. 2021; van Laar 2010). Although DNP and BNP are closely located and share similar environmental conditions, within the Boé region there is a wide network of rivers and water bodies surrounded by relatively well‐preserved gallery forests, which are used by chimpanzees to nest and feed (Binczik et al. 2017). Our results suggest that the Boé region (where BNP is located) is a stronghold for the chimpanzee population in GB. The effective protection and restoration of the natural habitats in ecological corridors connecting BNP and the remaining parks located south of the Corubal River (Figure 1b) could be beneficial to promote dispersal, potentially increasing gene flow and improving the probability for long‐term persistence of chimpanzees in coastal areas of GB, including the ones at CNP and CLNP.

### 4.3 Implications for the Estimation of *N*_*e*_ of Wild Populations of Primates

Here, we show that the estimated values of *N*_*e*_ using genomic data and more classic genetic markers, like microsatellite loci data obtained from non‐invasive fecal samples, are largely concordant, although we found that median *N*_*e*_ estimates produced by SNP data were higher than estimates generated using microsatellite data. This pattern was also reported by Clarke et al. (2024) meta‐analysis. Nonetheless, our study reinforces that datasets generated with traditional genetic markers, such as legacy or baseline microsatellite loci datasets for local populations, are of great value; these can be used to estimate parameters relevant to inform conservation management in species for which obtaining genomic data is not straightforward, such as wild‐born great apes, or in studies carried out in countries with limited access to sequencing units, funding, and trained researchers in genomic data (Bertola et al. 2024), such as GB (Ferreira da Silva et al. Forthcoming).

Moreover, our study shows that the combination of different molecular markers and analytical methods can be a useful strategy to overcome the limitations of obtaining high‐quality DNA from wild threatened populations, to investigate species' evolutionary history in time and space, and to integrate genetic information in conservation management decisions at local and regional scales.

## Disclosure

We declare that this work complies with the Convention on Biological Diversity and the Convention on International Trade in Endangered Species of Wild Fauna and Flora (CBD and CITES). Within the CBD, we followed the Access to Benefit Sharing (ABS) guidelines. We give credit and equal access to benefits to the countries involved in the study (Guinea‐Bissau, Portugal, and the UK) and the respective academic institutions and scientists involved in the collection and analysis of data, who are co‐authors of this work. We declare that we complied with CITES regulations and obtained export and import permits to move samples from Guinea‐Bissau to Portugal for analyses, following CITES guidelines.

## Ethics Statement

The manuscript has not been submitted elsewhere. The research complied with ethical guidelines, rules, and protocols approved by IBAP and CIBIO‐InBIO and adhered to the legal requirements of Guinea‐Bissau (GB) and Portugal. All except five samples were obtained non‐invasively from unidentified individuals without manipulation or perturbation of their daily behavior. Invasive samples (muscle and blood) were collected opportunistically from animals already deceased (muscle) or were collected during health checks of individuals living in captivity by a certified veterinarian (Dr. P. Melo, VetNatura). The blood collection was approved by IBAP. GB CITES focal point (Direção Geral de Florestas e Fauna) authorized the collection and exportation of blood and muscle samples. IBAP authorized the collection of fecal samples in protected areas and transportation to Portugal. ICNF Portugal (Instituto para a Conservação da Natureza e Florestas) and DGV (Direção Geral de Veterinária) authorized the importation of blood, muscle and fecal samples (Import Permits Simão blood sample 19PTLX00367, muscle sample run‐over individual N./No 18PTLX005871, and Bo, Bella, and Emilia blood samples 17‐PT‐LX00392/l).

## Consent

The authors have nothing to report.

## Conflicts of Interest

The authors declare no conflicts of interest.

## Supporting information


**Appendix S1:** eva70162‐sup‐0001‐AppendixS1.docx.

## Data Availability

The data that supports the findings of this study is available in Dryad Digital Repository (DOI: 10.5061/dryad.8sf7m0cz9 and DOI: 10.5061/dryad.j3tx95xpq).

## Endnotes

^1^
See documentary about rescuing chimpanzees to Ol Pejeta sanctuary, Kenya, https://www.youtube.com/watch?v=GxXMk2UPvUM.
