# Supplementary material for: Estimating the Effective Population Size Across Space and Time in the Critically Endangered Western Chimpanzee in Guinea‐Bissau: Challenges and Implications for Conservation Management
Source: Evol Appl. 2025 Oct 10;18(10):e70162. doi: 10.1111/eva.70162 (PMC12514012; doi:10.1111/eva.70162)
Supplement: Supplementary file 1 — Appendix S1: eva70162‐sup‐0001‐AppendixS1.docx. [file EVA-18-e70162-s001.docx]

**Supplementary material**

**Estimating the effective population size across space and time in the Critically Endangered western chimpanzee in Guinea-Bissau: challenges and implications for conservation management**

**Ferreira da Silva et al.**

**S1. Considerations on differences between the census size (*N_c_*) and *N_e_***

Although the concept of *N_e_* has been presented as analogous to the census size (*N_c_*), decades of research have shown repeatedly that *N_e_* and *N_c_* are not only distinct but may have nearly opposite trends under some models (Wakeley 1999; Mazet et al. 2016). We want to stress here that *N_e_* is only informative about some property of the genetic data, and different properties may have different temporal dynamics that are themselves possibly different from the *N_c_* dynamics (see Chikhi et al. 2018; Vishwakarma et al. 2024, Wakeley 1999). *N_c_* may also be very disconnected from any *N_e_* estimate because *N_c_* informs us about the current individuals living in the environment of interest and are thus directly affecting and affected by major ecological processes, such as predation, competition or density dependence (Waples 2022). Different *N_e_* values will depend on the probability that individuals have to contribute genes to the next generation but also on how populations may be connected to each other, as this will influence coalescence times (Beaumont 2004, Chikhi et al. 2010, Wakeley 1999). Contemporary *N_e_* will be influenced by recent fluctuations in population size, variance in reproductive success among individuals, unequal sex ratio and overlapping generations (Hoban et al. 2020, Waples 2022). Over longer time periods (“historical *N_e_*”), population structure and changes in connectivity may generate very contradictory results. For instance, Wakeley (1999) showed that a structured population where all the demes increase in size and between which gene flow increases at the same time, may exhibit a signal of decrease in *N_e_* in stark contrast with the increase of the actual population size. A similar phenomenon was described by Mazet et al. (2016) on the PSMC method (see also Parreira et al. this Special issue on the effect of population structure on different *N_e_* estimation methods). It is also important to note that, in the context of conserving threatened species, while *N_c_* may have direct relevance, the importance of effective population size *N_e_* is less immediately apparent.

**S2. Details on the production of the microsatellite loci dataset**

We generated a dataset of 143 unique genotypes for 10 microsatellite loci derived from non-invasive fecal samples (Borges, 2017, Gerini, 2018) (Fig. 1b). Eighty-five genotypes correspond to samples collected between 2015 and 2017 in CLNP (N=38), BNP (N=34) and DNP (N=13), and the remaining consisted of previously determined genotypes from CNP (N=58) (Sá, 2013) (Fig. 1b). Fecal samples were collected fresh and from unhabituated and unidentified individuals, in sites used by chimpanzee groups for sleeping, foraging and drinking. The techniques and methods to preserve the fecal samples until DNA extraction are described in Ferreira da Silva et al. (2014). DNA extraction was carried out using two methods: i) the QIAamp®DNA Stool Mini Kit (QIAGEN®) at the MWB research group laboratory facilities at School of Biosciences, Cardiff University, UK (Sá, 2013) and ii) the CTAB method (Vallet et al., 2008, adapted by Quéméré et al., 2010) for samples collected between 2015-2017, which were extracted at *Instituto Gulbenkian de Ciência* (IGC, Oeiras, Portugal) laboratory facilities. The procedures to avoid contamination by exogenous DNA are described elsewhere (Ferreira da Silva et al., 2014).

DNA samples were identified to the species level using a mitochondrial DNA hypervariable region I fragment (approximately 600 base pairs, using primers L15926 and H16555, as described in Sá, 2013). Consensus sequences were derived from forward and reverse sequencing by visual comparison using Geneious Pro v.4.8.5 (Biomatters, Biomatters Ltd, New Zealand). Standard Nucleotide BLAST in NCBI (<http://www.ncbi.nlm.nih.gov/>) was used to identify accessions closely related to the generated sequences and confirm that samples were from *P. troglodytes verus* (*i.e*., GenBank Accession code D38113). Allele size standardization between datasets was carried out using re-extraction and re-analyses of DNA extracts of five samples included in Sá (2013) together with the novel samples analyzed in Borges (2017) and Gerini (2018). Allele scoring followed previously described procedures to guarantee minimal impact of allelic dropout and false alleles errors: four replicates were carried out per sample and the rules to reach a consensus genotype were determined per locus (Ferreira da Silva et al., 2014). The consensus genotype was classified according to the Quality Index (QI, Miquel et al., 2006), and genotypes with a mean across loci below 0.55 were excluded from the dataset. The probability of identity (PI) and the probability of identity between siblings (PIsibs) (Waits et al., 2001), estimated using GenAIEx v.6.503 (Peakall & Smouse, 2006), was of 1.5 x 10^-11^ and 8.9 x 10^-05^, respectively, which in principle allows to distinguish between unique genotypes using six loci. We could not find genotyping errors (typing errors, large-allele dropout, and locus-specific deficiency in heterozygotes due to null alleles) using MicroChecker v.2.2.3 (van Oosterhout et al., 2006) apart from locus D2S1326 for CLNP, which showed excess of homozygotes. We retained the locus in the final dataset as we found no significant departures from Hardy-Weinberg equilibrium per locus using the Bonferroni correction when geographic populations were analyzed separately.

**S3. Summary of DNA extraction protocol used in blood and tissue samples (adapted from Vallet et al., 2008)**

The first day of DNA extraction started with adding warmed CTAB 2% solution to the samples after which a first lysis was performed at 60 ºC and 700 rpm for two hours. A second lysis was performed using CTAB 10% and incubating the samples at 60 ºC for two hours at 700 rpm. Proteinase K and chloroform-isoamyl were added to the samples and a precipitation step using cold isopropanol at -20 ºC was carried-out overnight. The second day of DNA extraction included two washes using cold ethanol 70%. DNA was eluted in TE. A cleanup was performed using pre-washed magnetic SPRI beads. To perform a preliminary quality control of the extractions, a 2% agarose gel was run, and DNA concentration was measured using Nanodrop. Laboratory procedures took place at the facilities of the *Instituto Gulbenkian de Ciência* (IGC), and extractions were carried out at a laminar flow hood in a Biosafety Level 2 dedicated room.

**Table S4. DNA concentration measured using Nanodrop**

| Sample | Concentration |
| --- | --- |
| Simao_PT_GB | 27,9 ng/µL |
| Bella_PT_GB | 53,9 ng/µL |
| Bo_PT_GB | 28,7 ng/µL |
| Emi_PT_GB | 48,0 ng/µL |
| T-3_Chimp | 13,2 ng/µL |

**Table S5. Whole Genome Sequencing (WGS) data summary statistics for each sample**

| Sample | Description | Coverage obtained | Observed number of homozygous genotypes | Expected number of homozygous genotypes | Number of non-missing genotypes | Inbreeding coefficient (F) |
| --- | --- | --- | --- | --- | --- | --- |
| Bella_PT_GB | obtained from blood that was conserved in EDTA and room temperature until DNA extraction | 16.373 | 3469972 | 3.723e+06 | 5557472 | -0.1381 |
| Bo_PT_GB | obtained from blood that was conserved in EDTA and room temperature until DNA extraction | 16.683 | 3488163 | 3.723e+06 | 5557411 | -0.1282 |
| Emi_PT_GB | obtained from blood that was conserved in EDTA and room temperature until DNA extraction | 18.769 | 3529060 | 3.723e+06 | 5557167 | -0.1058 |
| Simao_PT_GB | obtained from blood that was conserved in EDTA and room temperature until DNA extraction | 17.154 | 3457122 | 3.723e+06 | 5557101 | -0.145 |
| T-3_Chimp | Obtained from tissue (muscular) from a roadkill, found few minutes after accident. Sample was kept in 90% ethanol and room temperature until DNA extraction | 31.739 | 3425898 | 3.723e+06 | 5556748 | -0.1619 |

**Table S6. Initial values and varying sets of priors and hyperpriors, and respective variance of the mean (in brackets), tested using MSVAR 1.3 to reflect assumptions of stable populations (*N_0_* = *N_1_*), population declines (bottleneck, *N_0_* < *N_1_*), or moderate expansion (*N_0_* > *N_1_*). N_0_ – Current effective population size, N_1_ – Past effective population size, T – time of the inferred demographic change**

|  | | **Model 1**  **Stable population** | **Model 2**  **Bottleneck** | **Model 3**  **Severe expansion** | **Model 4**  **Moderate expansion** |
| --- | --- | --- | --- | --- | --- |
| **Priors** | **l**og10(N_0_) | 4 1 | 3 1 | 5 1 | 5 1 |
|  | log(N_1_) | 4 1 | 5 1 | 3 1 | 4 1 |
|  | log(Ɵ) | -3.5 1 | -3.5 1 | -3.5 1 | -3.5 1 |
|  | log(T) | 5 1 | 5 1 | 4 1 | 4 1 |
| **Hyperpriors** | **l**og(N_0_) | 6 2 0 0.5 | 4 1.4 0 0.5 | 4 2 0 0.5 | 3 2 0 0.5 |
|  | log(N_1_) | 5 2 0 0.5 | 4 1.4 0 0.5 | 5 2 0 0.5 | 5 2 0 0.5 |
|  | log(Ɵ) | -3.5 0.25 0 0.5 | -3.5 0.25 0 0.5 | -3.5 0.25 0 0.5 | -3.5 0.25 0 0.5 |
|  | log(T) | 5 2 0 0.5 | 5 2 0 0.5 | 5 2 0 0.5 | 5 2 0 0.5 |

**Table S7. Posterior distributions of the Median and 95% Highest Posterior density intervals (HPD; presented within brackets), calculated using BOA R package. Demographic parameters are as follows: *N_0_* - current effective population size; *N_1_* - past effective population size and T - time since the demographic change occurred in years. CNP – Cantanhez National Park, CLNP - Cufada Lagoons Natural Park, DNP - Dulombi National Park, BNP - Boé National Park**

|  |  | ***N_0_*** | ***N_1_*** | ***T*** |
| --- | --- | --- | --- | --- |
| ***GB*** | *Run 1* | 6 510 | 11 663 | 69 518 |
|  |  | (651 - 6,243,096) | (1060 - 302,134) | (31 - 140,637,132) |
|  | *Run 2* | 4 633 | 10 705 | 50 385 |
|  |  | (511 - 27,284) | (1435 - 95,675) | (106 - 56,728,330) |
|  | *Run 3* | 4484 | 11 910 | 54,388 |
|  |  | (606 - 23,889) | (1,215 - 256,980) | (208 - 140,087,698) |
|  | *Run 4* | 4411 | 11,026 | 44,596 |
|  |  | (532 - 29,134) | (1,208 - 153,462) | (85 - 132,647,784) |
| ***CNP*** | *Run 1* | 1,125 | 11,644 | 12,477 |
|  |  | (45 - 9,391) | (2,757 - 54,175) | (178 - 461,211) |
|  | *Run 2* | 1,021 | 10,637 | 10,879 |
|  |  | (50 - 9,080) | (2,553 - 44,668) | (237 - 360,081) |
|  | *Run 3* | 726 | 11,048 | 7,497 |
|  |  | (12 - 8,819) | (2,704 - 47,446) | (71 - 368,468) |
|  | *Run 4* | 566 | 10,615 | 5,288 |
|  |  | (8 - 7,518) | (2,540 - 44,056) | (59 - 219,685) |
| ***CLNP*** | *Run 1* | 1,225 | 11,416 | 7,874 |
|  |  | (17 - 16,885) | (2,532 - 51,523) | (64 - 587,895) |
|  | *Run 2* | 972 | 10,583 | 6,734 |
|  |  | (22 - 12,951) | (2,566 - 43,823) | (80 - 446,375) |
|  | *Run 3* | 647 | 10,950 | 4,759 |
|  |  | (8 - 11,051) | (2,719 - 45,394) | (42 - 219,281) |
|  | *Run 4* | 534 | 10,397 | 3,612 |
|  |  | (4 - 10,566) | (2,523 - 42,717) | (24 - 289,468) |
| ***DNP*** | *Run 1* | 7,401 | 9,543 | 13,110 |
|  |  | (70 - 367,113,200) | (390 - 2,669,931) | (4 - 627,769,194) |
|  | *Run 2* | 3,921 | 9,356 | 32,085 |
|  |  | (38 - 187,932) | (364 - 284,512) | (21 - 693,904,973) |
|  | *Run 3* | 3,642 | 10,651 | 24,992 |
|  |  | (13 - 1,236,517) | (348 - 4,369,181) | (8 - 757,355,877) |
|  | *Run 4* | 2,769 | 10,610 | 17,600 |
|  |  | (8 - 109,144) | (370 - 2,441,181) | (10 - 571,610,240) |
| ***BNP*** | *Run 1* | 24,643 | 8000 | 6,473 |
|  |  | (1,057 - 1,244,514,612) | (120 - 4,305,266) | (4 - 1,316,739,913) |
|  | *Run 2* | 7,925 | 7,320 | 47,435 |
|  |  | (295 - 856,446) | (104 - 316,082) | (7 - 1,201,434,227) |
|  | *Run 3* | 8,126 | 8,547 | 85,645 |
|  |  | (168 - 5,794,287) | (50 - 14,151,419) | (6 - 2,553,877,136) |
|  | *Run 4* | 6,716 | 8,555 | 75,875 |
|  |  | (43 - 1,085,425) | (55 - 13,696,197) | (5 - 2,552,701,303) |

**Figure S7** **Results for 10 bootstraps for each individual PSMC analyses**

Fig. S7 a) Bella_PT_GB

Fig. S7 b) Bo_PT_GB

Fig. S7 c) Emi_PT_GB


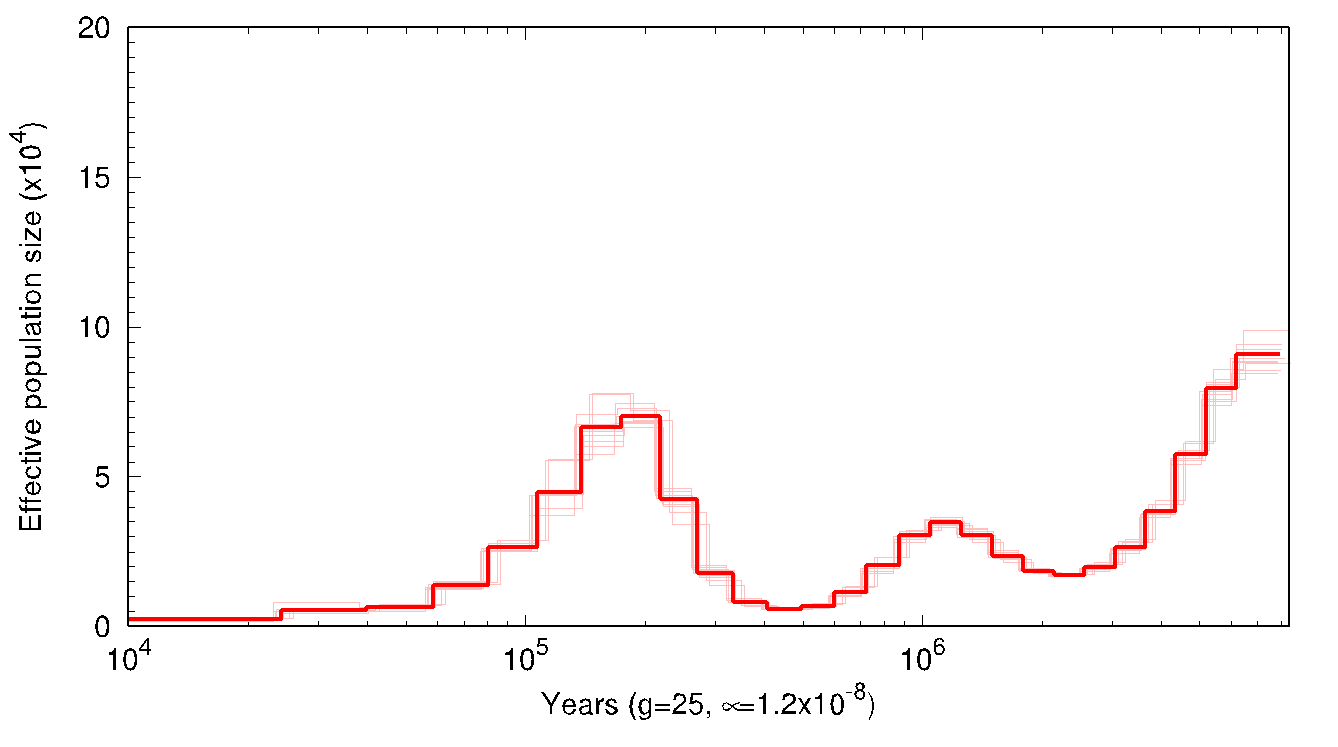


Fig. S7 d) Simao_PT_GB

Fig. S7 e) T-3_Chimp

Fig. S7 f) all samples
